# Ictères pathologiques du nouveau-né à l'hôpital Bonzola de Mbuji-Mayi, République Démocratique du Congo

**DOI:** 10.11604/pamj.2014.19.302.5658

**Published:** 2014-11-20

**Authors:** André Kabamba Mutombo, Olivier Mukuku, Benjamin Kasongo Kabulo, Augustin Mulangu Mutombo, Alain Mutombo Ngeleka, Junior Dibwe Mutombo, Maguy Sangaji Kabuya, Celestin Mukoko Kayembe, Oscar Numbi Luboya

**Affiliations:** 1 Département de Pédiatrie, Faculté de Médecine, Université Officielle de Mbuji-Mayi, RD Congo; 2 Département de Pédiatrie, Faculté de Médecine, Université de Lubumbashi, RD Congo; 3 Centre Hospitalier Pédiatrique de Mbuji-Mayi, RD Congo; 4 Département de Pédiatrie, Faculté de Médecine, Université de Kamina, RD Congo; 5 Ecole de Santé Publique, Université de Lubumbashi, RD Congo

**Keywords:** Ictère néonatal pathologique, fréquence, causes, traitement, évolution, Pathological jaundice, frequency, causes, treatment, évolution

## Abstract

L'ictère néonatal, bien qu'il soit souvent très banal, ne doit pas pour autant être négligé car il peut relever des étiologies variées et avoir des significations différentes au point de devenir pathologique. Au cours d'une étude rétro-prospective descriptive, les auteurs analysent les aspects épidemio-cliniques, thérapeutiques et évolutifs des ictères néonataux pathologiques à l'Unité des Urgences Néonatales de Bonzola à Mbuji-Mayi (RD Congo) sur une période de 5 ans. La fréquence est de 4,9% avec une incidence annuelle de 24 cas/an. La prédominance masculine est notée avec un sexe ratio de 1,1. Ces ictères sont précoces (31,1%) et tardif (17,5%). Les principales causes sont dans 81,6% des cas dominées par les infections (42,5%) et, incompatibilité fœto-maternelle (39,1%). La symptomatologie habituelle est notée: ictère franc et pâleur cutanéo-muqueuse (100%), urines jaunes foncées (62,5%), signes neurologiques (42,5%) et généraux (47,5%). Le traitement était à la fois étiologique (60%) et symptomatique ou d'appoint (40%) et a consisté dans l'association antibiotiques avec transfusion et/ou exsanguino-transfusion (24,2%), antibiothérapie seule (35,8%), inducteurs enzymatiques (35%) et photothérapie (5%). L’évolution a été bonne dans 82,3% des cas contre 9,3% des décès.

## Introduction

L'ictère néonatal est défini comme coloration jaunâtre des téguments et des muqueuses causée par un dépôt de bilirubine dans ces tissus [1]. Il est perceptible cliniquement dès que la bilirubine totale dépasse 70 mmol/L. C'est un signe clinique pratiquement constant chez le nouveau-né dans la première semaine de la vie et est souvent très banal [2]. Il est fréquent puisqu'il concerne 60 à 85% des nouveau-nés [3–5]. Il ne doit pas pour autant être négligé car il peut relever des étiologies variées et avoir des significations différentes au point de devenir pathologique. L’étiologie la plus souvent rencontrée est généralement l'hémolyse due à l'incompatibilité ABO. L'incompatibilité Rhésus (Rh), la déficience en glucose-6 phosphate déshydrogénase (G6PD), la polyglobulie, le céphalhématome, la septicémie, l'hypothyroïdie, les infections, les troubles métaboliques, les malformations congénitales, la prématurité, en sont les autres causes [6, 7]. Certains indices cliniques voire biologiques ne trompent pas. Ils doivent obligatoirement faire suspecter un ictère pathologique, c'est notamment: la survenue précoce avant 24 heures de vie; les signes d'hémolyse, les signes de choléstase; une durée supérieure à 10 jours; l'intensité de l'ictère allant jusqu'aux plantes de pieds [8–10]. L’étude des ictères néonataux a fait l'objet d'abondants travaux à travers le monde sous divers aspects. A Mbuji-Mayi (RD Congo), milieu de notre étude, aucun travail documenté n'a été consacré aux ictères en général et particulièrement aux ictères néonataux pathologiques. En vue de combler cette lacune, la présente étude est réalisée en vue de déterminer la fréquence de l'ictère néonatal ainsi que les aspects épidémiologiques, cliniques, thérapeutiques et évolutifs à l'Unité des Urgences Néonatales de l'hôpital Bonzola de Mbuji-Mayi en RD Congo.

## Méthodes

L’étude est descriptive, rétro prospective, transversale et monocentritique. Elle est réalisée sur une période de 5 ans (2007-2011) à l'Unité des Urgences Néonatales de l'hôpital Bonzola de Mbuji-Mayi en RDCongo. Elle a ciblé tout nouveau-né avec ictère pathologique hospitalisé pour prise en charge et répondant aux critères suivants: -un test de Coombs direct positif chez le nouveau-né avec ictère pathologique en dehors de tout contexte d'incompatibilité Rhésus ou de maladie auto-immune, incompatibilité de groupe (AO ou BO) étant prouvée [11–13]; le test de Munk Andersen concluant [14]; un examen somatique compatible avec une maladie hémolytique et une biologie confirmant une anémie de type hémolytique et/ou une érythroblastose [12, 13]. Tout cas d'incompatibilité ABO ne réalisant pas l'une des conditions d'inclusion précitées était considérée comme d’étiologie indéterminée. Les ictères simples ou physiologiques sont exclus de la présente étude. Est retenu comme ictère néonatal pathologique, tout ictère présentant des stigmates sémiologiques pré rappelées dans l'introduction. Les paramètres étudiés sont: -les aspects épidémio-cliniques: sexe, poids de naissance (réparti en <2500 grammes et ≥2500 grammes), période de survenue de l'ictère (très précoce s'il apparait avant le 3ème jour de vie, intermédiaire s'il survient entre le 3ème et le 7ème jour de vie et tardif s'il est noté après le 7ème jour de vie), symptomatologie, causes; les aspects thérapeutiques et évolutifs: traitement administré, issue du patient et séjour hospitalier. Le logiciel Epi-Info 2011 a été utilisé pour la saisie et l'exploitation des données, le calcul des moyennes et des fréquences (exprimées en pourcentage) ont été mis à contribution à cet effet.

## Résultats

### Fréquence- incidence des ictères néonataux pathologiques

Au cours de la période d’étude, 2410 enfants sont hospitalisés dans le service parmi lesquels 120 (le sont) pour ictère pathologique, ce qui représente une fréquence de 4,9% des admissions, pour une incidence annuelle de 24 cas par an.

### Aspects épidémio-cliniques

De ces 120 cas, 64 sont du sexe masculin (53,3%) et 56 du sexe féminin (46,6%). Le sexe ratio étant de 1,1 en faveur du garçon. Soixante-neuf d'entre eux (57,5%) pèsent plus de 2500 grammes contre cinquante et un (42,5%) dont le poids de naissance inférieur à 2500 grammes. La notion de souffrance péri et/ou néonatale est notée dans 12,5% des cas de notre série (15/120). L'ictère est très précoce dans 31,16% des cas, tardif dans 17,5% et intermédiaire dans 48,33% des cas. La symptomatologie est dominée dans plus de 60% des cas par:un ictère franc (100%), une pâleur cutanéo-muqueuse (100%), des urines jaunes foncées (62,5%). Les signes généraux (fièvre/ hypothermie), l'hépato-splénomégalie et les signes neurologiques surviennent respectivement dans 47,5%, 42,5% et 42,5% des cas. Les selles décolorées sont notées chez un seul patient (0,83%) (Tableau 1). Les causes inventoriées sont variées et reprises dans le Tableau 2 où l'infection et l'incompatibilité fœto-maternelle représentent les étiologies les plus fréquentes avec 81,6% des ictères néonataux pathologiques. Aucun cas de déficience en G6PD n'a été répertorié dans cette étude.

**Tableau 1:** Répartition des cas selon les caractéristiques épidémiologiques et cliniques

Paramètre	Effectif (n = 120)	Pourcentage
**Sexe**		
Masculin	64	53,4
Féminin	56	46,6
Sexe ratio	1,1	
**Poids de naissance**		
<2500 grammes	51	42,5
≥2500 grammes	69	57,5
**Antécédent de souffrance périnatale**	15	12,5
**Période d'apparition de l'ictère**		
Précoce (< J3)	41	31,2
Intermédiaire(entre J3-J7)	58	48,3
Tardif (>J7)	21	17,5
**Signes cliniques**		
Ictère franc	120	100
Pâleur cutanéo-muqueuse	120	100
Urines jaunes foncées	75	62,5
Fièvre /hypothermie	57	47,5
Hépato splénomégalie	51	42,5
Signes neurologiques	51	42,5
Selles décolorées	1	0,8

**Tableau 2:** Aspects étiologique

Cause	Effectif (n = 120)	Pourcentage
Sepsis	34	28,3
Incompatibilité fœto-maternelle BO	29	24,2
Incompatibilité fœto-maternelle AO	16	13,3
Méningite	15	12,5
Hépatite	1	0,8
Paludisme	1	0,8
Incompatibilité fœto-maternelle rhésus	2	1,7
Immaturité hépatique	5	4,2
Céphalhématome	5	4,2
Obstruction des voies biliaires	1	0,8
Indéterminée	11	9,2

### Aspects thérapeutiques et évolutifs

L'approche thérapeutique de nos ictères était à visée, à la fois étiologique (60%) et symptomatique (40%). Quarante-trois malades sur 120 (35,8%) ont bénéficié uniquement d'un traitement à base d'antibiotiques, 24 (20%) ont reçu l'association antibiotiques et transfusion, 5 (4,2%) étaient mis sous antibiotiques complétés par une exsanguino-transfusion. La transfusion seule a été utilisée dans 30% des cas et le phénobarbital était prescrit dans 35% des cas au début de la maladie avant d'adopter un des schémas thérapeutiques ci-dessus. Six malades (5%) ont subi des séances de photothérapie après une exsanguino-transfusion (Tableau 3). L’évolution a été, à court terme, bonne pour 99 nouveau-nés (82,3%). Nous avons déploré 11 décès dont 3 après exsanguino-transfusion. Dans 10 cas, l’évolution n'a pas été notée ou déterminée(les malades étant sortis contre avis médical ou ayant déserté ou sortis sur demande des parents) et pour lesquels une retro information n'a pu être obtenue. Le séjour hospitalier moyen était de 10 jours avec des extrêmes de 3 et 18 jours.

**Tableau 3:** Aspects thérapeutiques et évolutifs

Paramètre	Effectif (n = 120)	Pourcentage
**Traitement**		
AB seule	43	35,8
Phénobarbital	42	35,0
AB associés à la transfusion	24	20,0
Photothérapie	6	5,0
AB associés à l'exsanguino-transfusion	5	4,2
**Evolution**		
Bonne	99	82,30%
Décès	11	9,30%
Indéterminée	10	8,30%
AB : Antibiotique		

## Discussion

L'ictère néonatal pathologique constitue 4,9% des admissions néonatales au cours de la période d’étude avec une incidence annuelle de 24 cas par an (soit 2 cas/mois). Ces indicateurs épidémiologiques varient d'un auteur à un autre, d'un pays à un autre, d'une région à un une autre, d'une institution hospitalière à une autre voire d'une période à une autre. Cette fréquence est proche de 3,7% trouvé par Rabesandratana dans le service de néonatalogie à Mahajanga (Madagascar) [15] mais elle est largement inférieure à celles rapportées par Khatoon, Effiong, Barkat et Rasul qui sont respectivement de 35%, 32,6%, 26,3% et 22% [16–19]. Notre incidence annuelle est supérieure à celle trouvée à Kinshasa (RDCongo) aux Cliniques Universitaires en 1979 par Tady qui rapporte 12 cas par an [20]. La différence ainsi constatée s'expliquerait par des échantillonnages totalement différents, les types et milieu d’étude respectifs, les plateaux techniques de laboratoire (humain et matériel à la fois) des milieux concernés, des critères de sélection différents. Néanmoins, toutes les séries rapportées ont le mérite de souligner que les IN pathologiques demeurent un sérieux problème de préoccupation journalière des pédiatres dans leur milieu de travail à des degrés divers. Une légère prédominance masculine a été notée dans notre série avec un sex ratio de 1,1. Nos résultats sont superposables ou similaires à ceux rapportés par d'autres auteurs sans qu'une explication spécifique ne soit donnée [3, 11, 17, 18]. Quinze malades de notre série, spécifiquement les prématurés (12,5% des cas), ont présenté une souffrance péri et/ou néonatale. Ce constat a été fait également par Tady [20] et Laugier [12]. En effet, le rôle joué par l'anoxie/hypoxie voire la prématurité dans un contexte infectieux amplifié par les troubles hydro-électrolytiques et métaboliques associés doit être souligné et mis en exergue. Il est établi que dans le cadre de la toxicité de la bilirubine, la concentration de la bilirubine non liée peut être majorée par différents facteurs augmentant la perméabilité hémato-encéphalique(ou hémato-cérébrale) à la bilirubine ou déplaçant la bilirubine de l'albumine. C'est notamment la prématurité, l'infection, l'acidose, l'hypoxie, l'hypothermie, etc. [21, 22]. Ces facteurs peuvent aisément expliquer l'hyper bilirubinémie observée chez nos 15 malades ayant présenté une souffrance péri et /ou néonatale. Nous avons constaté que la date d'apparition de l'ictère reste un indicateur fiable permettant d'orienter vers un ictère pathologique. Plus il est précoce, intense, trainant ou plus il est tardif et prolongé, plus il impose le caractère pathologique. Ce constat a été fait et rapporté déjà par Laugier [12] et Tady [23]. Ce dernier rapporte des taux de 39,5% d'ictère précoce et de plus de 2% au delà de deux semaines de vie.

Les signes cliniques sont dominés par l'ictère franc et la pâleur cutanéo-muqueuse (100%), les signes généraux (47,5%), les urines jaunes foncées (62,5%), les signes neurologiques (42,5%). Tous ces signes en période néonatale sont en rapport avec une étiologie infectieuse [20, 24]. Ce constat corrobore celui fait par Tady à Kinshasa dans sa série où l'ictère franc et les signes neurologiques représentent respectivement 100% et 17% des cas [23]. Quant aux étiologies, les infections occupent la première place dans notre série, suivies des incompatibilités fœto-maternelles, loin devant l'immaturité hépatique, les céphalhématomes et l'obstruction des voies biliaires. Barkat trouve dans sa série que les étiologies des ictères néonataux pathologiques étaient dominées par les infections suivies des incompatibilités materno-fœtales [18]. Dans d'autres séries, c'est l'incompatibilité foeto-maternelle ABO qui vient en tête [4, 25]. Les travaux réalisés aux Cliniques Universitaires de Kinshasa par Tady donnent l'incidence de chaque étiologie de la manière suivante: incompatibilité fœto-maternelle dans 42% (dont 37% ABO), infections dans 23%, immaturité dans 6%, enzymopathie (déficit en G6PD) dans 5% et obstruction des voies biliaires dans 4% [20]. Les incompatibilités rhésus sont rares dans notre série et cette rareté serait due à celle du rhésus négatif dans notre milieu comme l'avaient confirmé déjà les travaux de Resseler en 1962 [26]. Cette faible incidence de l'incompatibilité Rhésus est aussi constatée dans l’étude de Alkhotani menée dans la région de Makkah qui rapporte que l'incompatibilité ABO et le déficit en G6PDsont les causes fréquentes de l'ictère néonatal alors que l'incompatibilité rhésus et la polyglobulie sont rares dans cette région [25]. Etant donné que les antibiotiques sont largement utilisés dans le cadre de toute suspicion d'infection néonatale, certaine ou probable (cas de méningite, sepsis ou d'autres infections à pyogènes), nous avons mis à contribution cette attitude dans notre série pour tous les cas dont l’étiologie n’était pas solidement établie ou en cas de suspicion d'infection comme repris au Tableau 3.

L'exsanguino-transfusion était préconisée et pratiquée selon les indications classiques [13, 27]. Contrairement à l'attitude de s'abstenir de procéder à une exsanguino-transfusion une fois que l'ictère nucléaire est constaté, car le traitement a peu d'utilité comme le soutiennent certains auteurs [24, 28], pour notre part, nous avons jugé que cette prise de position est trop rigide et préjudiciable pour le malade. En effet, nous avons opté de la faire quand même car d'une part, les lésions consécutives à un ictère nucléaire sont le plus souvent progressives et qu'au début de leur installation, l'exsanguino-transfusion peut en limiter l'extension (=effet bénéfique à exploiter en mettant tout du coté du malade); et d'autre part, il est établi que l'ictère nucléaire peut conduire rapidement à la mort si l'exsanguino-transfusion n'est pas entreprise ou réalisée à temps ou immédiatement [4]. C'est dans le souci de préserver la vie des malades et de limiter les dégâts que l'exsanguino-transfusion a été proposée dans le service tout en sachant qu'elle peut être ou serait sans effet sur les séquelles neurologiques préétablies (difficile à soutenir dans notre contexte de travail). Cette prise de position nous a permis de sauver quelques cas (2/5 des exsanguinés). L'effet essentiel recherché par cet acte est le remplacement de l'albumine plus ou moins saturée du nouveau-né par une autre provenant d'un adulte aux sites des liaisons disponibles avec pour résultat une épuration progressive de la bilirubine circulante (toxique) et une rééquilibration de la bilirubine entre les secteurs vasculaire et extravasculaire [29]. Cette exsanguino-transfusion a aussi un gros avantage de corriger l'anémie et d’éventuels troubles de l'hémostase, améliorant ainsi le transport de l'oxygène aux tissus. Le phénobarbital est prescrit dans le cadre des ictères par immaturité hépatique comme inducteur enzymatique dans le but de stimuler la glucuronyl transférase et la synthèse de la protéine Y du cytosol [11, 13, 30]. La photothérapie était aussi indiquée dans les ictères par immaturité hépatique et en plus comme traitement d'appoint après une exsanguino-transfusion.

## Conclusion

Les ictères d'origine infectieuse (41,5%) et par incompatibilité fœto-maternelle (39,1%) représentent l’étiologie la plus habituelle dans notre milieu. L'ictère est certes une pathologie fréquente, mais nous attirons l'attention sur l'importance de la mortalité et des séquelles neurosensorielles dans notre contexte en rapport surtout avec le non suivi des grossesses, les sorties précoces de maternité et les consultations tardives.
